# LncRNAs: key regulators and molecular mechanisms in lung cancer radiosensitivity

**DOI:** 10.1515/med-2026-1379

**Published:** 2026-03-06

**Authors:** Yuhan Chen, Jiajie Shi, Changcheng Qin, Shuhua Yang, Burong Hu, Junfang Yan

**Affiliations:** School of Public Health, Wenzhou Medical University, Wenzhou, Zhejiang, China; Zhejiang Engineering Research Center for Innovation and Application of Intelligent Radiotherapy Technology, The Second Affiliated Hospital of Wenzhou Medical University, Wenzhou, Zhejiang, China

**Keywords:** lncRNA, lung cancer, radiosensitivity, mechanism

## Abstract

Lung cancer presents a major global public health challenge due to its high incidence and mortality rates, highlighting the urgent need for effective treatment strategies. Radiotherapy, a cornerstone in lung cancer treatment, faces significant limitations due to both intrinsic and acquired radioresistance in tumors. Recent researches have identified long non-coding RNAs (lncRNAs) as critical regulatory factors that significantly affect the radiosensitivity of lung cancer cells. These lncRNAs are involved in key biological processes, including DNA damage repair, apoptotic signaling, cell cycle progression, tumor microenvironment remodeling. This review comprehensively summarizes and discusses the molecular mechanisms by which lncRNAs influence radiosensitivity and assesses their potential as predictive biomarkers for radiotherapy. Additionally, it analyzes the potential of lncRNA-based intervention strategies to overcome radioresistance and enhance radiotherapy regimens. The goal is to provide a robust theoretical foundation for advancing precision radiotherapy in lung cancer. In summary, lncRNAs function as multidimensional regulators of lung cancer radiosensitivity and represent promising targets for radiosensitization in the precision medicine era.

## Introduction

Long non-coding RNAs (lncRNAs) are defined as transcripts that exceed 200 nucleotides in length and lack protein coding potential [1]. Initially regarded as “junk RNAs” within the genome, recent research has revealed that lncRNAs serve as pivotal regulators of gene expression, influencing processes such as chromatin remodeling, transcription, and post-transcriptional modification [2]. Importantly, lncRNAs have emerged as significant contributors to the onset and progression of various diseases, including cancer [3], diabetes [4], and cardiovascular disease [5].

Lung cancer is the most prevalent cancer globally. According to the latest GLOBOCAN data, it accounts for 12.4 % of all cancer diagnoses, with approximately 2.4 million new cases reported annually. It is also the leading cause of cancer mortality among men in 89 countries [6]. According to statistics, the average 5-year survival rate for lung cancer patients is only 15–17 % [7]. Despite recent advancements in treatment, the survival rate for advanced lung cancer remains extremely low, highlighting the urgent need for more effective early diagnosis and personalized treatment strategies. Radiotherapy, one of the primary methods of treating malignant tumors, plays a significant role in lung cancer treatment. It is widely employed throughout various stages of lung cancer management, with approximately 70 % of patients recommended to receive radiotherapy at least once [8], 9]. However, various extrinsic and intrinsic factors contribute to radioresistance and diminish the therapeutic efficacy of lung cancer treatment. Intrinsic resistance arises mainly from tumor-intrinsic biological features, including DNA damage repair capacity, cell-cycle status, the proportion of stem-like cells, and specific gene mutations [10], 11]. These factors jointly determine the inherent sensitivity of tumors to radiation. Extrinsic resistance, by contrast, is imposed by the tumor microenvironment (TME), which comprises hypoxic regions, the vascular network, the extracellular matrix, and diverse immune cell populations. Radiotherapy not only directly kills tumor cells but also modulates immune responses. It can increase tumor immunogenicity or provoke immunosuppression, thereby reshaping TME composition and altering radiosensitivity of tumor [12], 13]. Consequently, a central goal of modern precision radiotherapy is to convert “resistant” tumors into “killable” ones by altering intrinsic resistance mechanisms and remodeling the TME.

Numerous studies have shown that lncRNAs contribute to lung cancer initiation and progression and exert a critical influence on tumor radiosensitivity. Prior reviews have summarized how lncRNAs modulate DNA damage repair and cell cycle progression to influence radiosensitivity of lung cancer [14], 15]. However, recent work increasingly highlights that lncRNA functions in radiation response extend well beyond classical DNA damage pathways, with particular relevance to remodeling of the TME [16], [17], [18]. Accordingly, this review focuses on how lncRNAs influence lung cancer radiosensitivity by regulating DNA repair, apoptosis, and cell cycle progression, and particularly emphasize their frequently overlooked but critical roles in TME regulation. Finally, we also assess the translational potential of lncRNAs as biomarkers and therapeutic targets in radiotherapy. By integrating fundamental mechanistic insights with clinical translation prospects, we aim to offer new perspectives for personalized lung cancer treatment and the optimization of radiotherapy strategies, ultimately enhancing patients’ prognosis and quality of life.

## Overview of lncRNAs

### The definition and basic characteristics of lncRNAs

LncRNAs represent a class of non-coding RNA molecules that exceed 200 nucleotides in length and are prevalent in eukaryotes. Despite lacking translational potential, they critically orchestrate gene expression, cellular differentiation, and disease pathogenesis through multifaceted mechanisms [19]. With advancements in high-throughput sequencing technologies, an increasing number of lncRNAs have been identified, confirming their functional diversity. According to the classification criteria established by the HUGO Gene Nomenclature Committee (HGNC), lncRNAs can be categorized into nine primary groups: long intergenic non-protein coding RNAs, microRNA non-coding host genes, antisense RNAs, small nucleolar RNA non-coding host genes, overlapping transcripts, divergent transcripts, intronic transcripts, lncRNAs with non-systematic symbol nomenclature, and lncRNAs designated under the FAM root naming system [20]. The secondary structure of lncRNA is complex and often forms specific spatial conformations, such as stem-loop structures, which are closely related to its functions. For instance, the LncDC tool significantly improves the classification accuracy of lncRNA and mRNA by integrating RNA sequence features, secondary structure k-mer scores, and flexible ORF features. Additionally, the expression of lncRNA exhibits high tissue specificity and may serve as a biomarker, especially under disease conditions such as tumor [21]. The evolutionary conservation of lncRNAs is relatively low, with most exhibiting species specificity, although their functional sequences or domains may retain a certain degree of conservation. Crucially, their spatiotemporal expression patterns under various stress (e.g., radiation exposure, oxidative damage) highlight essential roles in cellular homeostasis maintenance [22], 23].

### The functional mechanisms of lncRNAs

**Figure 1:**
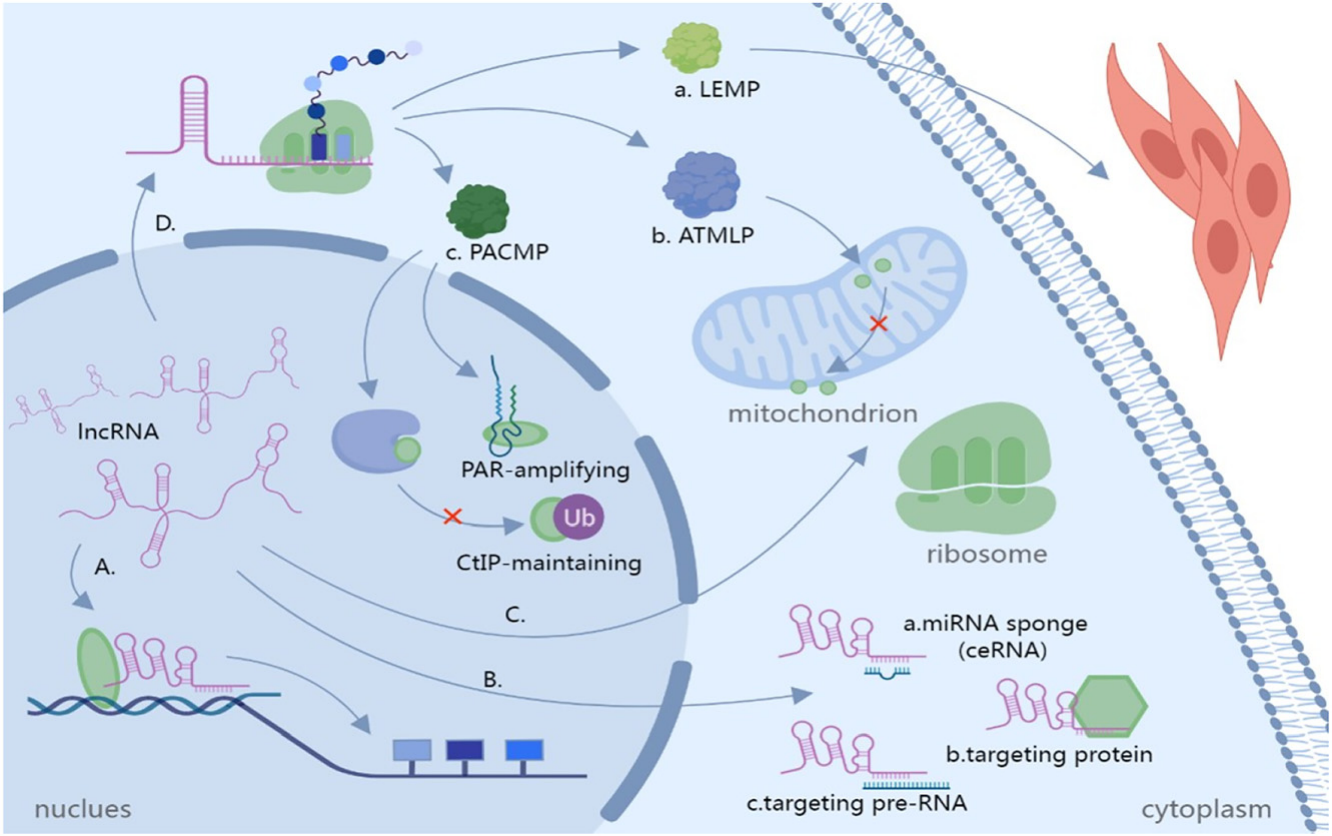
Subcellular localization and regulatory mechanisms of lncRNAs. (A) In the nucleus, lncRNAs function as signals, decoys, scaffolds, and guides. (B) Following transport to the cytoplasm, they perform diverse roles including: (a) Acting as ceRNAs to sequester miRNAs; (b) targeting proteins to form functional complexes that block or induce effects or alter localization; and (c) binding mRNA to modulate its stability, or translation. (C) A small subset of lncRNAs are transported into organelles to execute specific functions. (D) LncRNAs can also act indirectly by encoding micropeptides. LncRNA-encoded micropeptides execute specific roles: (a) ATMLP binds NIPSNAP1, inhibiting its inner-to-outer mitochondrial membrane translocation; (b) LEMP acts as an extracellular signal regulating muscle formation; and (c) PACMP inhibits CtIP ubiquitination in the nucleus while amplifying ADP-ribose signaling.

With the advent of high-throughput sequencing technology, the biological functions and mechanisms of lncRNAs in eukaryotes have been extensively elucidated, revealing their profound impact on diverse biological processes. A substantial body of literature supports the notion that lncRNAs can regulate gene expression and cellular processes by interacting with DNA, RNA, and proteins (Figure 1). These interactions occur through diverse mechanistic modes, including chromatin remodeling, transcription of both nearby and distant genes, RNA splicing and translation, as well as the formation and regulation of organelles and nuclear condensates [24], 25]. The functions of lncRNAs are closely related to their subcellular localization, and changes in localization may lead to their different roles within the cell [26]. In the nucleus, lncRNAs can serve various roles, including signals (e.g., Xist), decoys (e.g., GAS5, MALAT1), scaffolds (e.g., NEAT1), and guides (e.g., HOTAIR, linc-p21) [27]. In contrast, in the cytoplasm, lncRNAs regulate post-transcriptional fate and cellular signal transduction by influencing the stability and translation of associated mRNAs through interactions with RNA molecules. For instance, lncRNAs function as competing endogenous RNAs (ceRNAs), thereby regulating miRNA targets and their expression levels. Research has shown that the overexpression of PSMA3-AS1 competes with miR-101 for binding in NCI-H2170 cells, leading to an increase in the expression of EZH2, a critical histone methyltransferase [28]. This overexpression of EZH2 counteracts the inhibitory effects of PSMA3-AS1 knockdown on the proliferation, migration, and invasion of lung cancer cells. Additionally, some lncRNAs bind to mRNA and recruit specific proteins, shielding it from nuclease degradation, thus extending the mRNA’s half-life and enhancing protein synthesis. Research has shown that lncRNA SNHG1 acts as a molecular scaffold, recruiting HNRNPD protein to bind and stabilize SERPINA3 mRNA, thereby upregulating SERPINA3 protein expression and ultimately driving the migration and invasion of colorectal cancer cells [29]. Furthermore, lncRNAs can directly bind to specific proteins to alter their activities and function. For example, lncRNA Snhg6 has been identified as a regulator of myeloid-derived suppressor cell differentiation by modulating the ubiquitination of EZH2 [30]. Some lncRNAs are also transported into organelles to perform specific functions, such as lncRNA ZNFX1, which binds to the 40S ribosomal subunit to exert regulatory roles [31], and GAS5, which can translocate to mitochondria to maintain cellular energy homeostasis [32].

Although most lncRNAs are classified as non-coding RNAs, emerging evidences suggest that these molecules may contain short open reading frames capable of encoding micropeptides, which are small proteins comprising less than 100 amino acids. These micropeptides can undergo modifications and interact with other proteins to exert physiological or pathological roles [33] (Figure 1). For instance, micropeptides can localize to mitochondria and impact cellular metabolic processes. One example is lncRNA AFAP-AS1, which encodes a conserved 90-amino acid peptide known as the ATMLP. ATMLP binds to NIPSNAP homolog 1, hindering its translocation from the inner mitochondrial membrane to the outer mitochondrial membrane, thereby inhibiting cellular autophagy and lysosome formation. This, in turn, prevents the degradation of cellular contents, preserves cell viability, and repairs N6-methyladenosine (m6A) methylation-induced damages [34]. Additionally, micropeptides also regulate muscle regeneration, development, and contractile efficacy, such as LEMP encoded by lncRNA MyolncR4, which modulates myofibrillar formation through satellite cell activation and plays a pivotal role in skeletal muscle regeneration [35]. Furthermore, micropeptides are involved in the regulation of nuclear functions. A 44-amino acid peptide called the PAR-amplifying and CtIP-maintaining micropeptide (PACMP), encoded by lncRNA CTD-2256P15.2, prevents CtIP ubiquitination by inhibiting the formation of the CtIP-KLHL15 complex. It also directly binds to DNA damage-induced ADP-ribose chains, enhancing the activity of PARP1 and amplifying ADP-ribose signaling [36].

In summary, lncRNAs can interact with a variety of biomolecules including DNA, proteins, and RNAs to regulate gene expression and cellular processes. Their ability to encode micropeptides adds an additional layer to their functionality, enabling participation in fundamental biological processes such as epigenetic regulation, transcriptional regulation, and post-transcriptional regulation. These findings collectively underscore the significant biological and therapeutic implications of lncRNAs in both disease progression and treatment strategies.

## Role of lncRNAs in the development of lung cancer

### The correlation between lncRNAs expression and clinicopathological characteristics

Lung cancer pathogenesis is driven by a complex interplay of genetic alterations and dysregulated molecular pathways, among which lncRNAs have emerged as pivotal contributors to tumor initiation, progression, and metastasis. A comprehensive analysis of The Cancer Genome Atlas (TCGA) datasets by Wang et al. revealed 1,685 differentially expressed lncRNAs, with a striking predominance of upregulation (1,486 upregulated; 199 downregulated), underscoring their potential widespread oncogenic functions in lung cancer etiology [37]. These lncRNAs frequently correlate with pathological features, where elevated expression often predicts poorer prognosis, positioning them as promising therapeutic targets and biomarkers for early detection, diagnosis, and prognostication. For example, lncRNA LINC01614, which is secreted by cancer-associated fibroblasts via exocytosis and overexpressed in lung adenocarcinoma, promotes metabolic reprogramming. It directly interacts with ANXA2 and p65, triggering ANXA2-dependent p65 phosphorylation and acting as a scaffold to enhance NF-κB activation; thus, targeting CAF-specific LINC01614 could impede glutamine uptake and lung adenocarcinoma progression [38]. Similarly, lncRNA SOX2OT serves as a potent biomarker, exhibiting higher expression levels in lung adenocarcinoma tissues compared to normal tissues. Critically, low SOX2OT expression correlates with significantly improved overall survival, and its stability in body fluids and tissue-specificity make it viable for non-invasive screening and early diagnosis. This expression heterogeneity may clarify molecular subtypes, refining clinical strategies [39]. Advances in liquid biopsy underscore the translational potential of lncRNAs. For example, using this technique, exosome-carried MALAT1 showed high diagnostic accuracy, with the area under curve (AUC) value exceeding 0.85 [40]. Incorporating this lncRNA signal into a multi-analyte diagnostic panel is thus expected to improve early detection and molecular stratification of lung cancer.

### Roles of oncogenic lncRNAs in lung cancer progression and immune microenvironment

LncRNAs function as oncogenes through diverse mechanisms to drive lung cancer progression. In non-small cell lung cancer (NSCLC), their critical role is highlighted by reviews such as that by Ricciuti et al., which systematically catalogued 16 classic oncogenic lncRNAs, including HOTAIR, MALAT1, CCAT2, SOX2-OT, DLX6-AS1, and H19, that are central to regulating hallmarks of cancer such as sustained proliferation, evasion of apoptosis, and metastatic dissemination [41]. The oncogenic prowess of many lncRNAs extends beyond a single cancer type, underscoring their fundamental roles in malignant transformation. A paradigm is HOTAIR, the first lncRNA directly implicated in human cancers. It is consistently overexpressed in a wide spectrum of malignancies including colorectal cancer [42], breast cancer [43], 44], gastric cancer [45], cervical cancer [46], lung cancer [47], hepatocellular carcinoma [48], and pancreatic cancer [49], regulating processes like proliferation, apoptosis, invasion, and migration. In addition to carcinogenesis, lncRNAs are also crucial regulators of immune modulation within TME. Shuang Dai et al. comprehensively reported lncRNAs involved in key immune components in lung cancer, such as lymphocytes (e.g., NEAT1), cancer stem cells (e.g., HOTAIR, NEAT1, LINC01224, FOXF1-AS1, TCF7, LINC00662, DUXAP10, MCF2L-AS1, DANCR, and 20 other lncRNAs), macrophages (e.g., FGD5-AS1, XIST, GNAS-AS1, SOX2-OT), cytotoxic T lymphocytes (e.g., NKILA, NEAT1, SOX2-OT, SChLAP1), myeloid-derived suppressor cells (e.g., HOTAIRM1, RUNXOR, lncRNA PVT1, AK036396, MALAT1), regulatory T cells (e.g., LINC00301, C5orf64, NRK), and tumor vasculature (e.g., LINC00173.v1, EPIC1, LINC00667, MCM3AP-AS1, PVT1, LINC00312) [50]. Among these immune-regulating lncRNAs, NEAT1 is exceptionally significant due to its involvement in multiple immune processes.

### LncRNA-driven epigenetic regulation in lung cancer

LncRNAs function as key molecular hubs in lung cancer and interact extensively with diverse epigenetic mechanisms, thereby precisely modulating oncogenic signaling networks. For instance, in NSCLC, the significantly upregulated lncRNA CCAT1 is associated with poor survival and facilitates tumor progression by binding RAPTOR to induce AKT phosphorylation through interactions with USP49 and FABP5. Concurrently, it promotes the PI3K/AKT/mTOR pathway activation by facilitating FABP5 nuclear translocation, thereby enhancing the translation of the PPAR-RXR complex and the downstream kinase PDK1. This concerted action drives metabolic reprogramming and sustains the malignant phenotype of NSCLC cells, positioning CCAT1 as a central node in oncogenic signaling [51]. Similarly, ubiquitination represents another crucial epigenetic mechanism facilitated by lncRNAs in cancer. Specifically, the knockdown of lncRNA LINC00323 promotes the ubiquitin-mediated degradation of AKAP1 protein, and silencing LINC00323 inhibits NSCLC cell progression by downregulating AKAP1, highlighting a crucial lncRNA-protein stabilization axis in tumorigenesis [52]. The interplay between lncRNAs and various methylation modifications represents another critical layer of regulation. The stability and function of lncRNAs themselves can be controlled by m6A, the most prevalent internal mRNA modification in eukaryotes. For example, m6A modification enhances the stability of the oncogenic lncRNA SCIRT. This stabilized SCIRT promotes NSCLC progression by interacting with the splicing factor SFPQ and subsequently activating the PI3K/AKT pathway, illustrating how an epitranscriptomic mark can amplify lncRNA-driven oncogenesis [53]. Conversely, lncRNA expression is also controlled by DNA methylation at their promoter regions. The tumor suppressor lncRNA HCG11 is often silenced in lung adenocarcinoma through METTL14-mediated hypermethylation of its promoter. When expressed, HCG11 recruits the RNA-binding protein IGF2BP2 to stabilize and enhance the expression of the tumor suppressor LATS1 mRNA, thereby inhibiting tumor growth. Thus, the epigenetic silencing of HCG11 represents a loss of a critical growth-restraining mechanism [54]. In contrast, the oncogenic lncRNA LINC00857 is frequently overexpressed in lung adenocarcinoma due to promoter hypomethylation. Its dysregulated expression promotes tumorigenesis by acting as a molecular sponge for miR-486–5p, leading to the upregulation of its target NEK2, thereby identifying a novel methylation-dependent axis in lung cancer progression [55].

### Tumor-suppressor lncRNAs

Functioning as tumor suppressor genes, certain lncRNAs inhibit tumor development, whose loss or downregulation is a pivotal event in lung tumorigenesis. The restoration of their expression or activity represents a promising therapeutic avenue. Demonstrating this, Jin and colleagues found that lncRNA FTX expression is significantly downregulated in both NSCLC tissues and cell lines [56]; its upregulation leads to the inhibition of NSCLC proliferation and metastasis through FTX’s interaction with splicing proteins and subsequent activation of FOXA2 expression. Another layer of tumor-suppressive activity involves the direct targeting of core oncogenic signaling pathways. The TGF-β pathway, which plays a complex dual role in tumor suppression and promotion, is frequently exploited by cancer cells to drive epithelial-mesenchymal transition (EMT) and metastasis. The lncRNA GASL1 acts as a molecular counterbalance to this pro-carcinogenic shift. It is frequently downregulated in NSCLC tumors, and its re-expression has been demonstrated to suppress tumor growth by directly inhibiting the TGF-β signaling cascade, thereby hampering a critical driver of malignant progression [57].

Collectively, the aforementioned studies underscore the significant role of lncRNAs in regulating diverse cellular mechanisms driving lung cancer development. Consequently, lncRNAs present a promising array of biomarkers and therapeutic targets for tumor prevention, diagnosis, and treatment, highlighting their potential for clinical applications. In the realm of cancer treatment, researchers are exploring therapeutic strategies that incorporate lncRNAs. In the subsequent sections, we will delineate and discuss the specific mechanisms through which lncRNAs influence lung radiotherapy, thereby offering novel insights and strategies for targeting lncRNAs to enhance the efficacy of radiotherapy for lung cancer.

## The effect and mechanisms of lncRNAs on radiosensitivity of lung cancer

**Figure 2:**
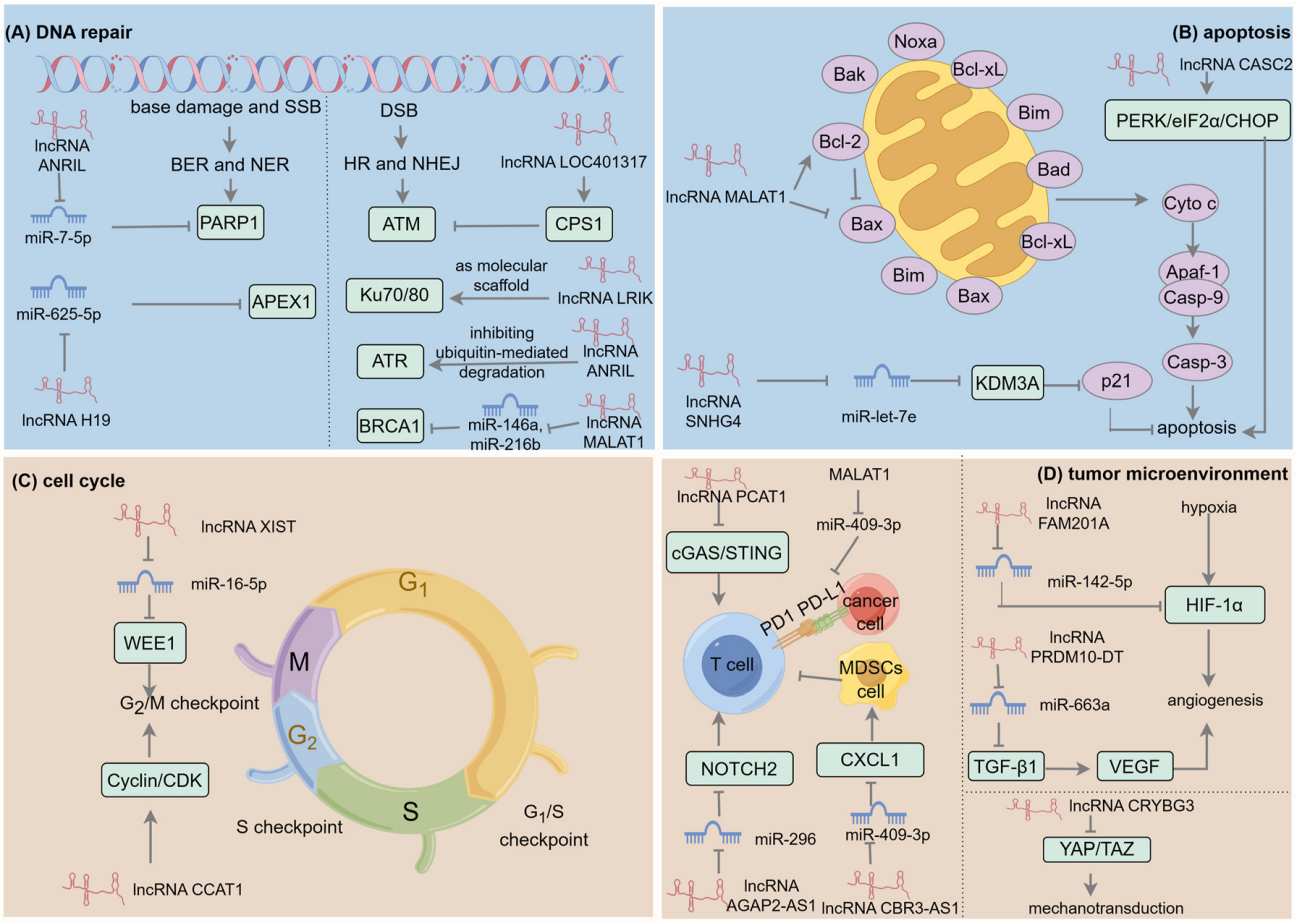
The mechanisms by which lncRNAs influence lung cancer radiosensitivity. (A) In DNA repair, lncRNA LOC401317 inhibits ATM activity by upregulating CPS1. LncRNA LRIK acts as a molecular scaffold that promotes NHEJ through interaction with the Ku70/Ku80 heterodimer. LncRNA ANRIL binds ATR and prevents its ubiquitination and degradation. ANRIL, H19, and MALAT1, sequester miRNAs to modulate the expression of PARP1, APEX1, and BRCA1. The resulting enhancement of repair-protein activity is a major contributor to radioresistance. (B) In radiation-induced apoptosis, lncRNA MALAT1 inhibits apoptosis by downregulating the pro-apoptotic protein bax and upregulating the anti-apoptotic protein Bcl-2. Conversely, lncRNA SNHG4 enhances apoptosis and radiosensitivity via the miR-let-7e/KDM3A/p21 axis. LncRNA CASC2 facilitates the apoptosis by activating the PERK/eIF2α/CHOP pathway. (C) Certain lncRNAs, such as lncRNA XIST and lncRNA CCAT1, influence cell cycle progression following radiation by regulating cell cycle-related proteins. (D) In immunity, the PCAT1/SOX2 axis suppresses T cell function by inhibiting the cGAS/STING pathway. Concurrently, lncRNA CBR3-AS1 recruits MDSCs through the ceRNA mechanism, further diminishing T cell activity. In contrast, lncRNA AGAP2-AS1 enhances T cell function by upregulating NOTCH2 via the ceRNA mechanism. Once activated, T cells can differentiate into cytotoxic T cells, which recognize and attack tumor cells, thereby enhancing the effectiveness of radiotherapy. In addition, MALAT1 increases PD-L1 expression by sequestering miR-140, which contributes to radioresistance. Hypoxia in the TME induces HIF-1α, which promotes tumor cell survival and stimulates angiogenesis, fostering radioresistance. LncRNA FAM201A can elevate HIF-1α levels by sponging miR-142-5p. LncRNAs also influence angiogenesis by modulating pro-angiogenic factors VEGF. Furthermore, radiation-induced lncRNA CCRYBG3 can inhibit mechanotransduction and increase radiosensitivity by suppressing the YAP/TAZ pathway.

The core mechanism of radiotherapy relies on inducing severe DNA damage through high-energy radiation to eradicate cancer cells. Importantly, the effectiveness of this treatment is highly dependent on tumor cell radiosensitivity, which is influenced by both intrinsic cellular and molecular characteristics of the cancer cells (intrinsic factors) and the external TME. Intrinsic factors encompass the genetic instability of cancer cells, which leads to critical molecular alterations that significantly enhance the cancer cell’s repair capacity, such as DNA repair and potentially lethal repair, or exhibit resistance to apoptosis characterized by abnormal apoptotic processes and dysregulated cell cycle mechanisms [58]. Therefore, a thorough understanding of the molecular mechanisms governing cancer cell radiosensitivity, combined with the identification of effective radiosensitizing targets, is crucial for enhancing radiotherapy efficacy. Accumulating evidence now establishes that lncRNAs significantly impact lung cancer radiosensitivity by regulating multiple key pathways central to vital cellular processes within tumor cells, as summarized in Figure 2.

### LncRNAs and DNA damage repair

Ionizing radiation can induce various types of DNA damage in cancer cells, including base damage, DNA double-strand breaks (DSBs), and single-strand breaks (SSBs) [59]. Cells address these lesions using a coordinated DNA damage repair network. Non-homologous end joining (NHEJ) and homologous recombination (HR) are the primary pathways for repairing DSBs. In contrast, base excision repair (BER) and nucleotide excision repair (NER) primarily manage simpler damages like base damage and SSBs [60], [61], [62]. Compared to normal cells, cancer cells exhibit heightened sensitivity to DNA damage due to potential mechanisms such as defects in the DNA damage repair pathway, increased replication stress, and elevated endogenous DNA damage [63]. However, through continuous evolution, cancer cells can mobilize complex signaling networks, including those orchestrated by lncRNAs, to compensate for repair deficiencies in one pathway, thereby enhancing overall DNA repair capacity and increasing radioresistance.

LncRNAs function as master regulators of the DNA repair, serving as molecular signaling hubs that precisely coordinate the detection, signaling, and repair of DNA damage (Figure 2A). They employ diverse mechanistic strategies to exert this control. For example, they can mediate the key DNA repair kinases. LncRNA LOC401317 significantly enhances the radiosensitivity of NSCLC cells by mediating the upregulation of CPS1 to modulate the core kinase, ATM, which orchestrates cellular responses to DNA damage through phosphorylation-dependent activation of cell cycle checkpoints and DNA repair factor recruitment [64]. Consequently, LOC401317 overexpression suppresses ATM signaling, ultimately enhancing NSCLC cell sensitivity to radiotherapy and demonstrating its potential as a regulator of DNA damage repair capacity. Acting as molecular scaffolds, some lncRNAs recruit repair proteins to damage sites, facilitating complex formation. For instance, lncRNA LRIK enhances NHEJ repair by interacting with the Ku70-Ku80 heterodimer [65]. Alternatively, lncRNAs stabilize key DDR proteins by inhibiting their ubiquitin-mediated degradation. A prime example is lncRNA ANRIL, which binds ATR (a critical kinase for HR repair of DSBs), preventing its ubiquitination and degradation, thereby sustaining ATR activity and HR efficiency [66]. Furthermore, lncRNAs function as ceRNAs, sequestering microRNAs (miRNAs) to relieve their repression of DDR effectors. ANRIL exemplifies this by acting as a sponge for miR-7-5p, consequently upregulating PARP1 and contributing to radioresistance. Targeting ANRIL thus holds promise for improving lung cancer radiotherapy outcomes [67]. Similarly, lncRNA H19 knockdown exacerbates radiation-induced DNA damage in A549 cells via the miR-625–5p/APEX1 axis, impairing BER by suppressing APEX1, which is essential for fixing base damage [68], 69]. MALAT1 also utilizes the ceRNA mechanism, sequestering miR-146a and miR-216b to upregulate BRCA1 expression and thereby protecting HR repair [70].

The above studies have revealed the bidirectional regulatory role of lncRNAs in regulating DNA damage signaling and repair. Certain tumor suppressor genes, including lncRNA LOC401317, increase the radiosensitivity of NSCLC cells by inhibiting crucial DNA repair proteins [64]. Conversely, lncRNAs such as H19 and MALAT1 function as oncogenes, enhancing the DNA damage repair and promoting radioresistance through various mechanisms [68], [69], [70]. However, the regulatory mechanisms of lncRNAs are complex and not fully understood, with individual lncRNAs sometimes exhibiting different effects across studies. Such differences may stem from the high heterogeneity inherent in cancer. Even within the same cancer type, such as lung cancer, there exist notable variations in genetic backgrounds, mutation profiles, and signaling pathway activities across different cell lines or patient samples. In addition, lncRNAs are intricately regulated by upstream signaling pathways and, in turn, influence numerous downstream target genes. Changes in any node within this network can significantly alter the functional outcomes of lncRNAs. Wu et al. conducted a systematic review of 87 studies, highlighting a key role of lncRNAs as ceRNAs that sponge miRNAs. The impact of this sponging depends on the specific miRNAs targeted. Consequently, the same lncRNA may yield different function depending on the cell type or environmental context it interacts with [71]. Therefore, to effectively translate lncRNAs into viable targets for radiotherapy sensitization, researchers should conduct comprehensive analyses of their specific regulatory networks across various molecular tumor subtypes.

### LncRNAs and apoptosis

As the primary mechanism of cell death following irradiation, apoptosis is significantly regulated by lncRNAs, primarily through their influence on key molecules within apoptosis-related signaling pathways (Figure 2B). For instance, lncRNAs can directly or indirectly regulate Bcl-2 family protein expression and thereby affecting the mitochondrial apoptosis pathway. Specifically, lncRNA MALAT1 suppresses radiation-induced apoptosis by inhibiting the pro-apoptotic protein Bax and upregulating the anti-apoptotic protein Bcl-2 [72]. Conversely, lncRNA SNHG4 acts as a molecular sponge for miR-let-7e, leading to increased levels of its target KDM3A. This diminishes p21 transcriptional activity, ultimately promoting apoptosis in NSCLC cells and enhancing their radiosensitivity [73]. Additionally, lncRNA CASC2 significantly increases the apoptosis rate in irradiated NSCLC cells by stabilizing PERK mRNA, thereby activating the PERK/eIF2α/CHOP endoplasmic reticulum stress pathway [74]. Collectively, these findings underscore the central regulatory role of lncRNAs in the apoptotic network and provide novel insights into the mechanisms underlying tumor cell resistance to radiation.

### LncRNAs and cell cycle

Cell cycle checkpoints (G_1_/S, S and G_2_/M) serve as critical nodes for maintaining DNA integrity and are key determinants of cellular radiosensitivity, with cells in G_2_/M phase being most sensitive, followed by those in G_1_/S phase, while S phase cells are most resistant to radiation [75], 76]. Studies have shown that lncRNAs can influence the processes of cell proliferation and apoptosis by regulating cell cycle-related proteins (Figure 2C). For instance, knockdown of lncRNA XIST enhances radiosensitivity in NSCLC cells; mechanistically, XIST represses miR-16-5p, thereby upregulating the expression of WEE1 [77], a critical G_2_ checkpoint kinase essential for the DDR at this checkpoint [78]. Similarly, inhibition of lncRNA CCAT1 increases sensitivity to radiotherapy, leading to G_2_/M phase arrest [79]. Together, these studies indicate that targeting lncRNAs in radiotherapy to inhibit key cell cycle proteins, such as the checkpoint kinase WEE1, can induce arrest in the G_2_/M phase, which is especially radiosensitive. At G_2_/M phase, DNA replication is complete and chromatin becomes highly condensed, reducing accessibility for repair machinery and lowering repair efficiency. This impaired repair promotes mitotic chromosome catastrophe and subsequent cell death, thereby increasing cellular radiosensitivity [80]. Therefore, modulating lncRNAs to alter checkpoint kinase activity and provoke cell cycle arrest, especially at G_2_/M, represents a promising radiosensitization strategy.

DNA damage repair, cell cycle arrest, and apoptosis are integral cellular responses to ionizing radiation stress. Collectively, these processes form the DNA damage response (DDR) [81]. Inhibitors targeting DDR pathways can enhance cellular radiosensitivity by disrupting DNA repair and cell cycle progression. For example, PARP inhibitors such as Olaparib and Niraparib target the PARP family. PARP1, a central DDR enzyme, primarily mediates repair of SSBs. By inhibiting PARP activity, PARP inhibitors prevent SSB repair, causing these lesions to convert into DSBs. Tumor cells with BRCA1/2 mutations or HR deficiencies are especially vulnerable to this effect, producing a synthetic lethal interaction [81], [82], [83]. Additional targets within the DDR network include core damage repair, cell-cycle regulators such as DNA-PK, ATR, ATM, CHK1 and WEE1 [84], [85], [86].

However, the clinical efficacy of DDR inhibitors is often limited by acquired resistance, which arises from adaptive tumor cell responses and signaling pathway reprogramming [81]. Consequently, multi-target combination strategies are gaining attention. Incorporating lncRNA-targeted agents may reverse this resistance by simultaneously inhibiting DDR components at the protein and non-coding RNA levels. Such dual targeting is expected to increase DNA damage accumulation, promote tumor cell apoptosis, and potentiate radiotherapy. Preliminary cell and animal studies have confirmed that combining DDR inhibitors with lncRNA-targeting approaches can enhance radiosensitivity. In mouse tumor models, inhibiting lncRNA AK144717 or DDUP enhances ATM-dependent DDR inhibition, significantly suppresses tumor growth, and increases the efficacy of radiotherapy [87], 88]. Furthermore, the combined strategy of inhibiting key DDR proteins alongside micropeptides encoded by relevant lncRNAs demonstrates a synergistic effect, effectively suppressing tumor cell proliferation and promoting the accumulation of DNA damage [36].

Clinical investigation remains at an early stage, and the safety and efficacy of combination regimens require additional validation. To date, clinical practice has concentrated primarily on single DDR inhibitors and their combination with radiotherapy, while targeted therapies against lncRNAs have not yet entered widespread clinical trials. As the understanding of lncRNA functions and their interaction mechanisms with DDR inhibitors deepens, these combination treatment strategies are anticipated to undergo clinical evaluation. This progression will likely advance the development of personalized radiotherapy-enhanced treatments.

### LncRNAs and tumor microenvironment

#### Immunity

Radiotherapy serves as a cornerstone of cancer therapy not only through direct tumor cell killing but also by profoundly reshaping the tumor immune microenvironment, where dynamic changes critically influence treatment efficacy and patient prognosis. Radiotherapy induces immunogenic cell death, triggering the release of damage-associated molecular patterns and tumor antigens that activate both innate and adaptive immunity [89]. This cascade promotes dendritic cell maturation and antigen presentation, subsequently enhancing effector T cell activation to generate an “*in situ* vaccine” effect [90]. Conversely, radiotherapy can simultaneously initiate immunosuppressive responses, such as polarizing tumor-associated macrophages (TAMs) towards the M2 phenotype while increasing infiltration of myeloid-derived suppressor cells (MDSCs) and regulatory T cells (Tregs), thereby dampening anti-tumor immunity [91]. This bidirectional relationship extends to immune modulation of radiosensitivity itself: immunosuppressive cells like M2-TAMs and Tregs typically promote radioresistance, whereas CD8^+^ T cell infiltration and M1 macrophage presence correlate with heightened tumor cell radiosensitivity [92].

LncRNAs critically shape tumor radiosensitivity by modulating immune cell infiltration and cytokine secretion within the TME (Figure 2D). In NSCLC, the PCAT1/SOX2 axis suppresses T cell immunity by inhibiting the cGAS/STING signaling pathway, thereby promoting tumor proliferation. Notably, combining radiotherapy with inhibition of this axis reactivates cGAS/STING signaling, triggering potent immune responses that significantly enhance radiotherapy efficacy [16]. Similarly, lncRNA AGAP2-AS1 regulates the TME through ceRNA mechanism: it sequesters miR-296, leading to NOTCH2 upregulation, a signaling pathway critical for B cell and T cell development, cytokine secretion, and immune function, positioning it as a promising target for radioimmunotherapy [17], 18], 93]. Another key player, lncRNA CBR3-AS1, employs ceRNA activity to bind miR-409–3p, inducing CXCL1 expression. This recruits MDSCs while suppressing T cell activity, a process mediated by methyltransferase RBM15 via m6A-IGF2BP3-dependent pathways. Critically, RBM15 depletion synergizes with radiotherapy to suppress NSCLC growth, highlighting its therapeutic potential [94]. Conversely, the MALAT1/miR-140 axis contributes to radioresistance by inducing G_2_/M arrest via PD-L1 regulation, facilitating DSB repair and promoting tumor cell survival [95]. Collectively, these findings elucidate how lncRNAs reshape the TME through diverse immune and cellular pathways, revealing novel targets for optimizing radiotherapy-immunotherapy combinations.

#### Hypoxia, angiogenesis and mechanotransduction

Hypoxia refers to the phenomenon of insufficient oxygen supply in tumor tissues, typically caused by the irregularity and inefficiency of the tumor vascular network. It is a hallmark of solid TME and a major driver of radioresistance, critically impairing treatment efficacy and patient prognosis [96], 97]. At the molecular level, hypoxia stabilizes HIF-1α and prevents its proteasomal degradation, which triggers broad transcriptional reprogramming. Once activated, HIF-1α initiates adaptive responses that promote tumor cell survival, induce angiogenesis, and drive metabolic changes like glycolysis, collectively forming a formidable barrier against radiotherapy [97]. LncRNAs are crucial regulators in the HIF-1α signaling pathway (Figure 2D). For example, in NSCLC, the lncRNA FAM201A functions as a molecular sponge for miR-142-5p, relieving this miRNA’s translational repression of HIF-1α mRNA. This post-transcriptional upregulation of HIF-1α protein enhances hypoxia adaptation and increases tumor cell resistance to radiotherapy [98]. LncRNAs widely modulate tumor-cell responses to hypoxia, creating a complex regulatory network. For instance, in cervical cancer, lncRNA UCA1 is transcriptionally activated by HIF-1α and enhances the Warburg effect by upregulating HK2, a key glycolytic enzyme. By boosting glycolysis, UCA1 supplies energy to tumor cells under hypoxia and sustains their proliferation, which indirectly contributes to radioresistance [99]. Likewise, in nasopharyngeal carcinoma, lncRNA PVT1 stabilizes HIF-1α mRNA and establishes a positive feedback loop with HIF-1α, thereby continuously amplifying hypoxic signaling [100].

Angiogenesis, frequently hyperactivated in tumors by signals like HIF-1α, provides essential nutrients that fuel tumor growth and metastasis [101]. Cancer cells that survive after radiotherapy may acquire blood supply through angiogenesis, thereby enhancing their invasive capabilities. For instance, radiation can indirectly activate EMT and glycosylation, promoting cancer cell migration [102]. This angiogenic switch represents a key mechanism of radioresistance, a concept validated clinically by the improved efficacy observed when combining anti-angiogenic drugs (e.g., Sorafenib) with radiotherapy in hepatocellular carcinoma [103]. LncRNAs critically modulate this process by regulating pro-angiogenic factors like VEGF, thereby influencing tumor oxygenation and radiation response (Figure 2D). A key example is PRDM10-DT, an X-ray-induced pro-angiogenic lncRNA that functions as a ceRNA to sequester miR-663a. This sequestration relieves miR-663a-mediated suppression of TGF-β1, activating the TGF-β1/VEGF signaling axis to drive angiogenesis, metastasis, and ultimately radioresistance [104].

Furthermore, mechanotransduction, a fundamental regulator of TME dynamics, critically influences cellular structure and function. Building on this, irradiation-induced upregulation of lncRNA CRYBG3 inhibits mechanotransduction processes, specifically affecting downstream Hippo signaling pathway effectors YAP/TAZ, reshaping the cellular mechanical perception and response, and ultimately inhibiting the survival of lung cancer cells (Figure 2D). This reveals the convergence of physical and biochemical signals at the lncRNA node [105].

In summary, lncRNAs multi-dimensionally regulate the TME to influence lung cancer radiosensitivity. Their mechanisms exhibit remarkable complexity across diverse domains including immune cell function, hypoxia adaptation, angiogenesis, and mechanotransduction. Future research should prioritize delineating precise lncRNA-TME interaction networks to advance precision radiotherapy-immunotherapy combinations. Concurrently, research should explore combination therapy strategies that integrate lncRNA targets with immune checkpoint inhibitors, hypoxia modifiers and anti-angiogenic drugs. Additionally, employing imaging techniques to monitor dynamic changes in tumor oxygenation could lead to more accurate efficacy assessments. These efforts will improve assessment of treatment response and offer new approaches to overcome radioresistance driven by immunosuppression, hypoxia, and angiogenesis.

## LncRNAs as biomarkers and therapeutic targets for radiosensitivity in lung cancer

Abundant evidences confirm that specific lncRNAs demonstrate significant potential as biomarkers for radiotherapy, predicting patient treatment response and prognosis [106], 107]. However, a single lncRNA biomarker has inherent limitations and cannot fully capture the heterogeneous response of tumors to radiotherapy. Consequently, recent efforts have focused on screening and constructing multi-lncRNA signatures to improve risk stratification and prognostic accuracy for radiotherapy. For example, combined-signature models centered on CASC19 and LINC01977 have shown promising clinical potential. Using radiotherapy data from NSCLC patients in the TCGA database, investigators identified four lncRNAs, CASC19, LINC01977, LINC02471, and MAGI2-AS3, that associate significantly with radiotherapy response and used them to build a risk-scoring model [108]]. The model reliably discriminates radiotherapy-sensitive from radiotherapy-tolerant patients and demonstrates strong prognostic performance. Functional assays further indicate that silencing LINC01977 markedly reduces radiotherapy tolerance in NSCLC cells, supporting its central role in modulating radiosensitivity. On the other hand, cuproptosis, a newly identified mode of copper-ion-dependent programmed cell death, holds significant biological importance, particularly in tumor development, with a notable impact on lung cancer. In this context, cuproptosis-related lncRNAs demonstrate potential for enhancing the prognostic evaluation of lung cancer radiotherapy. A study focusing on NSCLC developed a risk model incorporating six cuproptosis-related lncRNAs: AC104088.1, PPP4R3B-DT, AC006042.3, LUCAT1, HHLA3-AS1, and LINC02029. Within this model, LUCAT1, HHLA3-AS1, and LINC02029 are identified as high-risk lncRNAs (Hazard Ratio>1), while the others serve as protective lncRNAs (Hazard Ratio<1). The model’s predictive capability was validated through various analyses, including survival analysis, risk curve assessment, independent prognostic tests, and ROC curve evaluation, demonstrating superior predictive performance compared to traditional clinical indicators like age, gender, and pathological stage [109]. Additionally, some studies have enhanced the accuracy and comprehensiveness of prognostic evaluations by integrating genes associated with multiple cell death modes, such as apoptosis, ferroptosis, and necrosis [110], 111].

The combined signature of multiple lncRNAs also links closely to the tumor immune microenvironment. Constructing lncRNA regulatory networks has shown that radiotherapy-associated lncRNAs influence immune cell infiltration and contribute to tumor immune escape after radiotherapy, thereby offering potential biomarkers and targets for combined immunotherapy [108]]. Advanced imaging approaches, such as pulmonary MRI with different gas agents, have further expanded noninvasive options for lung cancer diagnosis and prognosis monitoring [112], 113]. Integrating lncRNA signatures with these imaging techniques improves prediction of radiotherapy response and supplies a molecular rationale for individualized treatment planning [114].

Although lncRNAs hold great promise as biomarkers, their clinical translation faces several challenges. One major issue is the heterogeneity and stability of lncRNA expression, which restricts their reliability as biomarkers. Expression levels can vary significantly among patients, cancer subtypes, and even within different regions of the same tumor. Systematic reviews and meta-analyses reveal that while many lncRNAs are linked to radioresistance, their expression trends are inconsistent across various cancer types and sample sources, limiting the reproducibility and generalizability of findings [115]. Additionally, most existing studies rely on small sample sizes, lacking the large-scale and systematic clinical validation needed for broader application [108]. In terms of detection technology, qRT-PCR has emerged as the dominant method for detecting lncRNA expression in clinical samples due to its high sensitivity and ease of use. However, it requires high-quality RNA samples and is not suitable for high-throughput screening. In contrast, RNA sequencing offers a comprehensive lncRNA expression profile and can identify new biomarkers, but it is expensive and requires advanced data analysis skills [116]. Digital PCR, an emerging absolute quantification technology, addresses the limitations of traditional PCR by eliminating the need for internal references and enhancing the detection of low-abundance lncRNAs [117]. Nonetheless, the low concentration and intricate structure of lncRNAs in body fluids present technical challenges [118]. The integration of CRISPR/Cas13a with photoelectric detection has enabled highly sensitive dual lncRNA detection, enhancing diagnostic accuracy and offering innovative approaches for non-invasive clinical testing [119]. Despite these advancements, these technologies face challenges such as high costs, complex procedures, and demanding data analysis requirements, hindering their widespread clinical adoption.

LncRNAs not only participate in gene expression regulation but also significantly influence tumor radiosensitivity and resistance, making them promising targets for combined radiotherapy and targeted therapy. Strategies to enhance radiosensitivity by targeting lncRNAs primarily involve molecular interventions like antisense oligonucleotides (ASOs), which promote target degradation through specific binding [120], 121], as demonstrated by the efficacy of MALAT-1 ASOs in preclinical cancer models [122], and small interfering RNAs (siRNAs) delivered via nanocarriers (such as PVT1-targeting liposome complexes) that enhance esophageal cancer cell radiosensitivity [123]. In addition, CRISPR technology can also be employed for site-specific knockout or regulation of the lncRNA genome [124]. These studies indicate that ASO, siRNA, and CRISPR interventions centered on lncRNAs exhibit significant radiosensitizing effects *in vitro* and in animal models. This suggests that lncRNAs are promising targets for adjuvant radiotherapy. However, the clinical application of these technologies faces challenges, including delivery efficiency, tissue specificity, and off-target effects [125]. To address these issues, researchers are developing innovative delivery systems, such as viral vectors and nanoparticles, to enhance treatment efficacy and minimize side effects [126], 127]. Concurrently, a deeper understanding of the molecular mechanisms of lncRNAs will aid in identifying more effective targets and advancing their clinical translation.

## Conclusion and future perspectives

In this review, we systematically analyze and discuss the diverse roles and mechanisms by which lncRNAs influence the onset and progression of lung cancer, with particular emphasis on their modulation of tumor radiosensitivity. Our analysis reveals that lncRNAs regulate radiotherapy response not only via canonical DDR pathways, but also through dynamic remodeling of the TME, including immune regulation, hypoxia adaptation, angiogenesis, and mechanotransduction. These multifaceted roles underscore the potential value of lncRNAs for precision radiotherapy.

However, the roles of lncRNAs in lung cancer radiotherapy are complex and varied. Many experimental studies show that specific lncRNAs modulate radiosensitivity or radioresistance and that their expression correlates with patient prognosis, indicating promise as biomarkers. However, some studies report divergent functions and regulatory networks for the same lncRNAs, so caution is warranted when interpreting and applying these findings to avoid oversimplifying their biological roles. Large-scale validation that integrates multi-omics data with clinical samples will help resolve these discrepancies and support more reliable clinical translation of lncRNA findings.

Currently, lncRNAs not only serve as molecular markers for predicting lung cancer patients’ responses to radiotherapy but also reveal regulatory mechanisms that offer new therapeutic targets for developing radiotherapy sensitizers and overcoming resistance. For instance, by targeting specific lncRNAs, researchers aim to impair the DNA damage repair capacity of tumor cells and modulate the immune microenvironment, potentially boosting radiotherapy efficacy. Nevertheless, despite extensive basic research, the clinical translation of lncRNAs remains sluggish. Key challenges include their tissue-specific expression and functional diversity, coupled with the absence of efficient and safe delivery systems [125]. Moreover, standardizing and individualizing the molecular diagnosis and treatment of lncRNAs in clinical practice remains an urgent issue that needs resolution.

Future research should focus more on understanding the specific mechanisms of action of lncRNAs. By integrating emerging technologies like single-cell sequencing and spatial omics, researchers can comprehensively map the regulatory networks and dynamic changes of lncRNAs associated with lung cancer radiotherapy [128]. Concurrently, multi-center and large-sample clinical validations should be enhanced to facilitate the translation of lncRNA-related biomarkers into clinical applications. Furthermore, advancing innovative RNA interference technologies, nanocarriers, and gene-editing tools will provide robust technical support for lncRNA-targeted therapies [129]. Through interdisciplinary collaboration, establishing a complete pathway from basic research to clinical application is anticipated to achieve precision management in lung cancer radiotherapy.

In conclusion, lncRNAs play a pivotal role as regulators in lung cancer radiotherapy, offering extensive opportunities for research and application. By systematically balancing various research outcomes and delving into their molecular mechanisms, while also aligning with clinical practice needs, we can foster innovative strategies in lung cancer radiotherapy. Looking ahead, precise diagnosis and personalized treatment based on lncRNAs are poised to greatly enhance treatment efficacy and improve the quality of life for lung cancer patients, ushering lung cancer radiotherapy into a new era of advancement.
